# Effective image visualization for publications – a workflow using open access tools and concepts

**DOI:** 10.12688/f1000research.27140.2

**Published:** 2021-02-18

**Authors:** Christopher Schmied, Helena Klara Jambor

**Affiliations:** 1 Leibniz-Forschungsinstitut für Molekulare Pharmakologie im Forschungsverbund Berlin e.V. (FMP), Berlin, Germany; 2 Mildred-Scheel Early Career Center, Medical Faculty, Technische Universität Dresden, Dresden, Germany

**Keywords:** Image Publication, FIJI, Good principles of figure design, Beginner's workflow, Image processing, open source, Visualization, Image analysis

## Abstract

Today, 25% of figures in biomedical publications contain images of various types, e.g. photos, light or electron microscopy images, x-rays, or even sketches or drawings. Despite being widely used, published images may be ineffective or illegible since details are not visible, information is missing or they have been inappropriately processed. The vast majority of such imperfect images can be attributed to the lack of experience of the authors as undergraduate and graduate curricula lack courses on image acquisition, ethical processing, and visualization.

Here we present a step-by-step image processing workflow for effective and ethical image presentation. The workflow is aimed to allow novice users with little or no prior experience in image processing to implement the essential steps towards publishing images. The workflow is based on the open source software Fiji, but its principles can be applied with other software packages. All image processing steps discussed here, and complementary suggestions for image presentation, are shown in an accessible “cheat sheet”-style format, enabling wide distribution, use, and adoption to more specific needs.

## Introduction

Every year, about 800,000 articles are newly indexed at Pubmed ( https://www.nlm.nih.gov/bsd/index_stats_comp.html) of which 25% contain images ^1^; this amounts to about 500 new articles with image figures per day. These published images provide new insights, but each day also the number of problematic images increases. While intentionally manipulated images are rare ^2, 3^, erroneous handling of images is more common. Another problem is that methods often insufficiently inform about image acquisition and processing ^4^. Last, images frequently have low legibility, as only 10–20% of published images provide all key information (annotation of color/inset/scale/specimen) ^5^. In the long run, problematic images may undermine the trust in scientific data and, when published in emerging image archives, reduce the value of such repositories ^6, 7^.

Today’s scientists face rapidly evolving technologies and employ many methodologies, with microscopy and image analysis ^8^ just one among many. Problematic images thus partially arise from: 1) lack in training, as ethical and legible processing of microscopy data is not systematically taught ^4^, 2) lack in local expertise, as image facilities are restricted to a few research hubs, and 3) while publishers established guidelines for handling image forgeries ^9– 11^, actionable and clear instructions for legible image publishing are lacking.

Here, we introduce an image processing workflow to effectively and ethically present images. The step-by-step workflow enables novice users, with no image processing experience and occasional microscopists, with no intention towards specializing in image processing to take the first steps towards publishing truthful and legible images.

## Methods

Obtaining high quality bioimages starts with specimen preparation such as fixation, labelling and clearing. To acquire and resolve the biological structure of interest, choose a microscopy system with an objective lens that allows suitable resolution, optical sectioning and spatial sampling. It is vital to sample intensity information properly by choosing a sufficient bit depth and avoiding saturation of high intensities. If the microscope-system allows changing the detector offset, low intensities should not be cut off. Rather than down sampling and cropping the image data, choose an appropriate magnification. When possible, align or rotate the sample to avoid image rotations. For comparison of image data, sample preparation and image acquisition settings need to be the same ^12– 19^.

**Figure 1.**
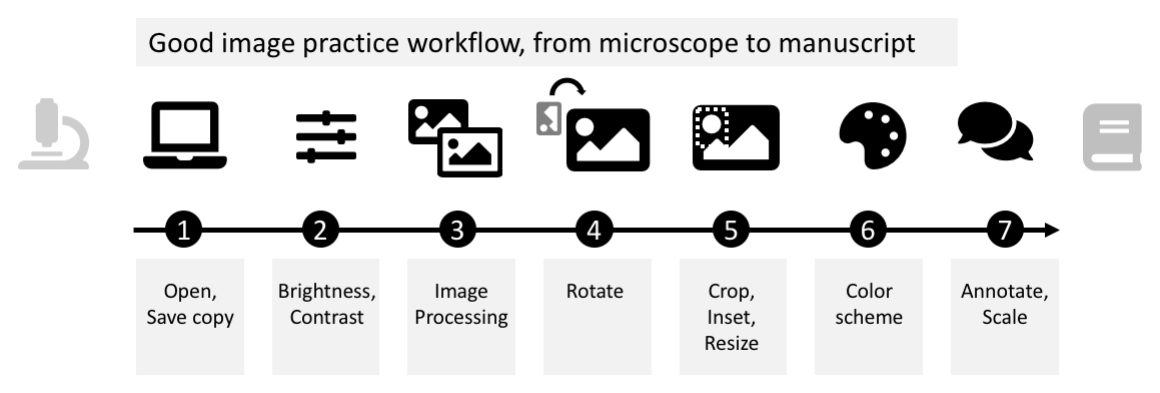
Schematic of the image processing workflow from microscope to manuscript.

After acquisition, bioimages can be processed and prepared for publication using the workflow below ( Figure 1), which is visually summarized in cheat-sheet style ( Figure 3 and Figure 4). Both are based on Fiji ^20^, an open source, free image analysis program for bioimages. Images are quantitative data. While image visualizations allow qualitative assessments, it is important to accompany them with quantitative measurements and appropriate statistical analysis. This workflow strictly addresses the image processing necessary for presentations and figures. Images prepared for presentation (e.g. 8-bit, RGB) are unsuitable for subsequent quantification such as intensity measurements. We therefore recommend separating image quantification and visualization workflows. Finally, documentation of any imaging and image processing workflows is key for reproducibility ^21^.

### Step 1: Open & save

Duplicate the raw image to retain the original, untouched image as raw data and only process the duplicate. Load image-duplicate into Fiji and make sure **metadata** (see Table 1: Glossary), such as the **scale**, are correct. When possible use the **Bio-Formats** plugin for import, as this reads key image metadata (e.g. scale) automatically along with the image ^22^.

**Table 1.** Glossary.

**Bio-Formats**	Software tool by the Open Microscopy Environment (OME). Aims to read the most common open source and proprietary bioimage data formats. Saves in the open OME-TIFF format, preserving image data along its metadata.
**Bit depth**	Range of intensities i.e. number of gray values in an image 8-bit: 256 gray values; 16-bit: 65,536 gray values
**Brightness & contrast** **- Intensity adjustment**	Computer screens display only 8-bits per RGB channel and 8-bits for grayscale images. The brightness / contrast setting is a transfer function between the intensity information in the image and its display. Linear and non-linear transfer functions exist (see also gamma correction). NOTE: the displayed range of the image is further restricted by our limited visual perception.
**Compression**	Aims to reduce file sizes and can be either lossless or lossy. Lossless methods allow to rebuild all the original image values, whereas lossy compression achieves bigger and/or faster size reduction accepting some loss of information.
**DPI**	Printers produce dots on paper. Dots per inch, DPI, specifies the used resolution.
**False color LUTs**	Instead of translating gray values to a linear range between black and white one can also use different colors or nonlinear ranges. Such a visualization can also conceal details or information in an image and needs to be clearly stated.
**Gamma correction**	Makes some intensity ranges in an image more visible while reducing the visibility of others. Uses a nonlinear transfer function, its shape is adjusted by the gamma value. This nonlinear change is not obvious and thus needs to be clearly stated.
**Gray value**	Specific intensity value in an image (see bit depth).
**Histogram** **equalization**	Images are highly variable in their gray value distribution with some gray values occurring more often than others do. This makes inefficient use of the very restricted bit depth for visualization. ‘Histogram equalization’ redistributes the gray values giving a bigger visible range to gray values that occur more often and reducing the range for less occurring gray values. This nonlinear change needs to be clearly indicated.
**Image histogram**	Visualizes the gray level distribution of an image. It plots the number (count) of pixels over each gray level (or specified gray level bins) present in the image.
**Interpolation**	E. g. if one increases the number of pixels in an image the values of the newly creates pixels needs to be computed, this happens using interpolation algorithms.
**JPEG**	A lossy compression standard resulting in a .jpg or .jpeg file. Compression is always applied when generating a JPEG file and repeated opening and re-saving will increase the loss of information.
**LUT**	In an image, the Look up Table (LUT), translates specific numeric values into shades of gray or color to then be displayed on a screen or on paper.
**Metadata**	Additional information such as physical dimension (see scale) or formation of an image e.g. microscope, objective lens.
**PNG**	PNG file formats use a lossless compression to store a single image (no movies or stacks) with up to 24-bit in RGB or 16-bit grayscale. All metadata such as pixel size is lost, overlays are burned into the image data and brightness / contrast settings are applied. For final use in presentations or figure assembly in tools such as illustrator or Inkscape.
**RGB image**	Image composed of a red, green and blue channel.
**Scale**	Each pixel represents a sample at a defined physical space of the imaged object. The scale relates the pixels to this physical dimension.
**TIFF**	TIFF formats can handle multiple dimensions (stacks, channels, frames) and stores the raw image data in a container. TIFF files also include information about the image content (e.g. dimensions, pixel size) and can contain other information such as a region of interest. Bioimage analysis software usually stores TIFF files, which preserve the original image data information; some image metadata (e.g. microscope, objective etc) may be lost. Store intermediate results in TIFF.

When processing is complete, several options exist (see glossary): saving images in **TIFF** format preserves the entire information. TIFF files however can rarely be properly used in programs for figure assembly (e.g. Inkscape, PowerPoint). For image presentation (figures, slides, online), save images in **PNG** format, which irreversibly merges the image with annotations, permanently applies brightness/contrast settings, and saves multiple channels as 24-bit **RGB image**. Another common image presentation file format is **JPEG**, which should be rarely used due to its lossy **compression** ^19^. Beware of incorrect or unintentional **bit depth** conversions ^23^.

### Step 2: Brightness & contrast

Images with a large **gray value** range may appear black when opening them in FIJI ^12^. To properly display such data for the purpose of presentation/communication ^24^, adjust the **brightness and contrast**. For comparisons of intensities across images, it is recommended to use the same fixed intensity values (‘set’). For adjustments, avoid auto-buttons as, depending on the software packages, the underlying code may differ, resulting in display differences. Linear **intensity adjustment** is acceptable, as long as key features are not obscured and minimal background signal is still visible to provide audiences with a sense for signal specificity. Entirely eliminating the background signal, or completely ‘clipping’ high intensities, is misleading (see also ^9, 19, 25^). Some saturated pixels in the image are acceptable, if this helps the visualization. To identify problems with intensity sampling, or seeing if the image has been processed, the **image histogram** can be used to show its gray value distribution ( Figure 3). Briefly, good unprocessed images should have some offset in the low intensity range ( Figure 3: **Histogram A**). The distribution should not be cut off in the high range ( Figure 3: **Histogram B**) and the range should be continuous ( Figure 3: **Histogram C**). 

Non-linear adjustments of brightness and contrast, for example **histogram equalizations** or **gamma correction** must be explained in both figure legend and method section ^19, 26, 27^. Miura and Nørrelykke nicely describe why intensity adjustments (linear and non-linear) must be applied with special caution when images have already been pre-processed, e.g. cropped ^21^. Once images have been adjusted, ‘apply’ and ‘save as PNG’ irreversibly change the intensity range, which makes images unsuitable for intensity measurements. 

### Step 3: Image processing

Depending on your specific scientific question and goal, further image processing may be necessary for image visualization. For instance, advanced systems such as lightsheet microscopy require extensive image processing workflows to obtain a reconstructed volume of the biological specimen for visualization ^28– 32^. Large 3D volumes of data are also hard to visualize in two dimensional figures and require the use of projection or rendering ^33^. Finally, microscopy systems add artefacts (noise, blur), which image processing using linear filters ^13^ and deconvolution ^34, 35^ can help to reduce. Any processing for representing the image data needs to be carefully applied where necessary and is no replacement for an optimized imaging setup ^12– 18^. The processing needs to be clearly stated in the methods section, advanced or non-linear adjustments also in the figure legends ^13, 19^.

### Step 4: Rotation & resizing

Image rotations are sometimes necessary to compare image content properly. For instance, when comparing specimens, it helps to align them in the same anatomical orientation. Image rotations however result in a redistribution of the intensity values within the fixed image pixel grid: for rotations by less than 90 degrees, new intensity values are computed by **interpolation**, and thus information is lost ( Figure 3). For rotations in multiples of 90-degree steps, pixels can be reordered rather than interpolated, however this depends on the specific implementation of the rotation algorithm ( Figure 3). Loss of information by image rotation may be acceptable for image visualizations, however all image quantifications should be done beforehand ^19, 26^.

### Step 5: Cropping

Often larger fields-of-view are captured on the microscope than are required in the figure. Cropping is then not only permissible, it is necessary to focus the reader on the relevant result. In contrast, it is not ethical to crop out data that would change the interpretation of the experiment, or to “cherry-pick” data ^9, 19, 26^. We discourage adjusting the intensity of individual crops especially for comparisons ^21^. When a larger field of view and a magnification of detail (‘inset’) need to be shown side-by-side, indicate inset position in the original image. Adjust the size of the image in the figure preparation software, not during image processing: Image size adjustments by upsampling or downsampling an image, requires interpolation and thus may degrade image quality.

### Step 6: Color

In fluorescence microscopy, cameras usually capture each wavelength (channel) with a separate grayscale image. Here, no signal is shown as black, and intensities of the fluorescent signal are displayed in steps of grey values with saturated pixels shown in white. When only one fluorophore/wavelength/channel is shown in a figure, grayscale, which has the best contrast, is favorable. Consider also inverting the grayscale images as human brightness perception is logarithmic and can best differentiate bright areas ^27^. Inverted grayscale images are also printer-friendly and have better visibility on a white page/slide. To visualize several channels of a specimen (e.g. colocalization studies), encode channels with different colors. A look-up table ( **LUT**) determines how gray values are translated into a color value. Additionally, we see at times the use of **false color LUTs** for visualizing image data; when used improperly, false color LUTs can be highly misleading ^27^ and therefore should be explicitly mentioned in methods and figure legends.

### Step 7: Annotate

Images represent physical dimensions and can depict different scales ranging from nanometer to millimeter, which is often not obvious ^36^. Adding scale information, ideally a scale bar with dimension, onto or next to the image, therefore is essential for self-explanatory figures. Also annotate what each color and symbol represents in an image, again best in the image itself or next to it. The aim is to provide sufficient information to the reader to understand the presented result at a glance. Ensure that scale bar, dimensions and annotations are legible in the final figure to be published; it may be more time efficient to adjust scale bar and add dimensions/annotations in the figure preparation software (e.g. as described here ^37^).

### Testing of workflow

We tested the workflow on fluorescently-stained microscope images of Drosophila egg chambers (RRID:BDSC_5905; ^38^) and the HeLa (RRID:CVCL_0030) ImageJ sample image ^39^. For generating a “poor” image example, we processed the raw microscope images minimally, only converting the bit depth from 16-bit to 8-bit and retained default color schemes. We did not add annotations, performed no image cropping, rotation, or specific brightness contrast adjustments as these often lack in poorly visualized images ^5^. We thus simulated images as they are typically “processed” in the majority of current publications ^5^. To perform a qualitative assessment, we tested image visibility to color blind (deuteranopia) audiences using the color blindness simulator (RRID: SCR_018400; ^40^).

## Results

**Figure 2.**
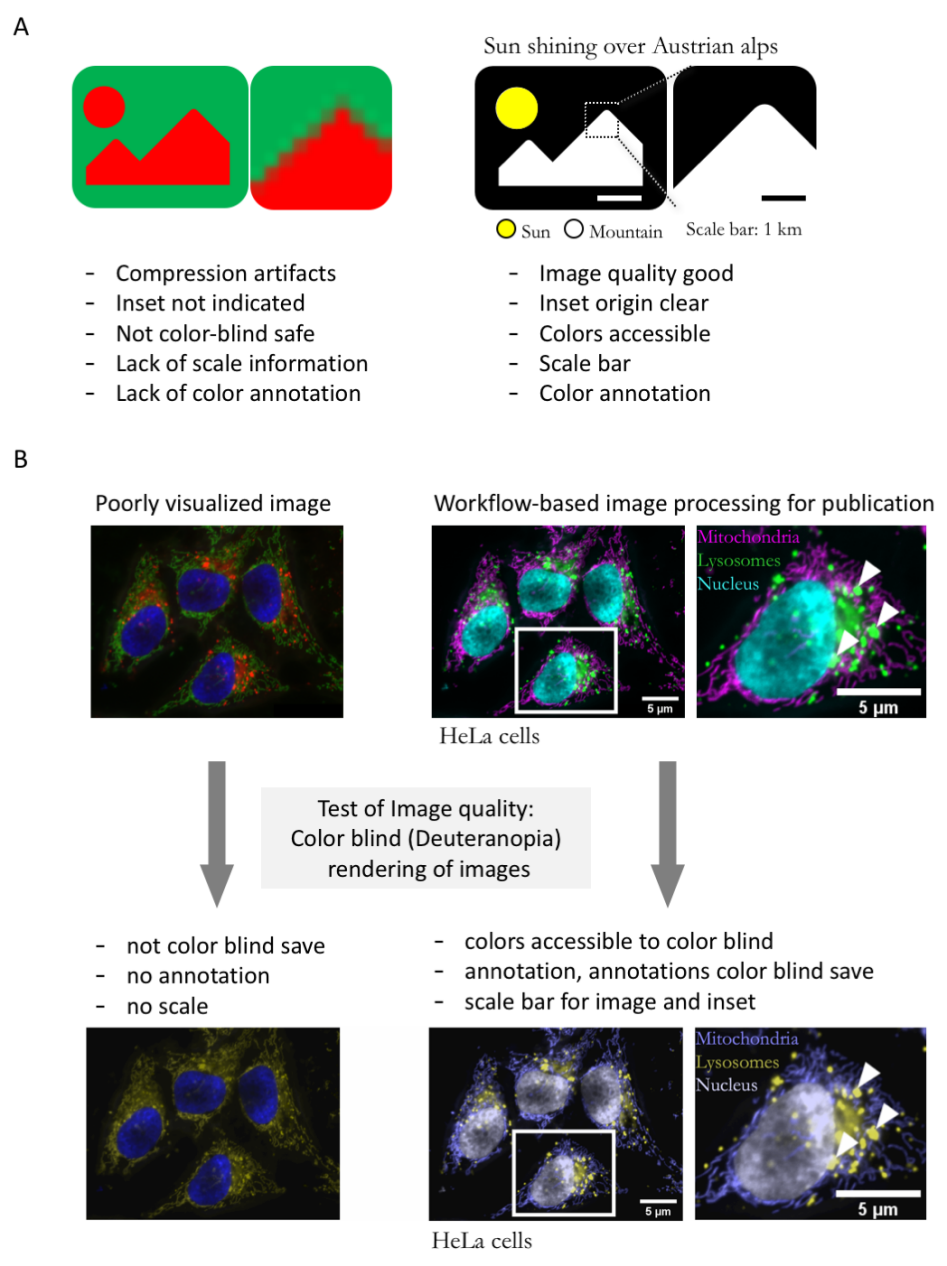
**A**. Schematic of typical errors in published bioimages and improved version of exemplary image without compression artifacts, and with accessible color-code, annotation, and scale. **B**. Poorly visualized example image, image after processing with the workflow presented here, and test of image accessibility to color blind readers.

Using our example microscope images, we qualitatively compared the readability of images processed with or without the workflow described (schematic: Figure 2A). Images for which the steps of the workflow were implemented contained the key information, were cropped to maximize focus, and sufficiently annotated (color channels, scale, organism), while images processed minimally without following the workflow have a “poor” readability ( Figure 2B). As further example of readability, we also demonstrated that images processed according to our workflow are accessible to color blind readers ( Figure 2B).

**Figure 3.**
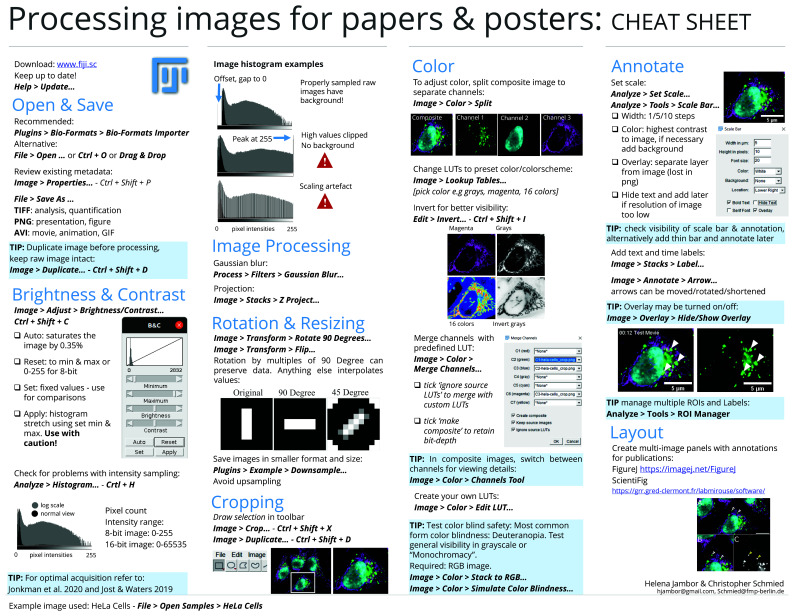
Cheat sheet 1: processing images for papers and posters ^45^.

**Figure 4.**
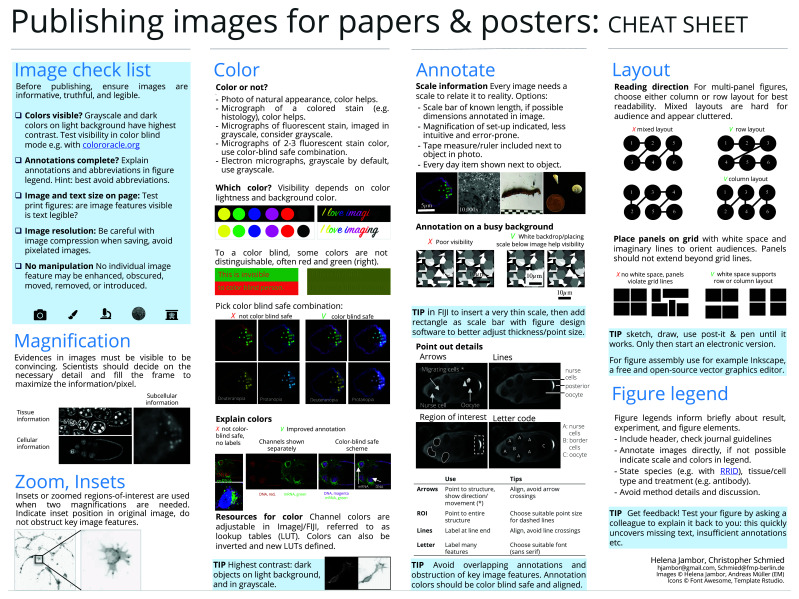
Cheat sheet 2: publishing images for papers and posters ^45^.

The workflow steps and accompanying suggestions for image presentation are available as accessible “cheat sheets” ( Figure 3 and Figure 4) for wide distribution and adoption to more specific needs. Our workflow is based on the open source software Fiji ( Figure 3), but its principles ( Figure 4) are applicable to other software.

After completing the workflow, images may be assembled for publication and legends added ^41^. Layouting images on a page can be done with design software such as the free and open source Inkscape ( https://inkscape.org) or the proprietary Adobe Illustrator. Several options also exist to prepare publication-ready figures directly in ImageJ/FIJI, for example ScientiFig and FigureJ ^42, 43^. Figure resolution is usually referred to as dots per inch ( **DPI**). For an ‘unpixelated’ display of microscopy images in an electronic publication, publishers require 300 DPI images in RGB color mode. (Note that the dots-per-inch do not correspond to the physical dimension of the microscopy object and scale bar but solely refer to image size in print or on the screen). This workflow is iterative and feedback from colleagues helps to identify possible hurdles.

## Conclusion

If followed, the workflow helps avoiding common problems of published 2D images, but principles are also applicable to 3D stacks and movies. Indeed, lack of truthful scientific communication and reproducibility are among the biggest problems faced by science today ^44^ and considering that an estimated 500 publications with images are published daily, improving image quality could have a profound impact in tackling this issue.

## Data availability

### Underlying data

HeLa cell test images are available at: https://imagej.nih.gov/ij/images/hela-cells.zip. *D.melanogaster* egg chamber cells images are available on Open Science Framework.

Open Science Framework: Effective image visualization for publications – a workflow using open access tools and concepts. https://doi.org/10.17605/OSF.IO/DF3MQ ^45^.

### Extended data

Open Science Framework: Effective image visualization for publications – a workflow using open access tools and concepts. https://doi.org/10.17605/OSF.IO/DF3MQ ^45^.

This project contains the following extended data:

**-** Processing_images_cheatsheet_SchmiedJambor.png (printable image of cheat sheet 1)
**-** SchmiedJambor_Figures3_Cheatsheet1.eps (modifiable version of cheat sheet 1)
**-** Publishing_ images_cheatsheet_SchmiedJambor.png (printable image of cheat sheet 2)
**-** SchmiedJambor_Figures4_Cheatsheet2.eps (modifiable version of cheat sheet 2)

Data are available under the terms of the Creative Commons Zero “No rights reserved” data waiver (CC0 1.0 Public domain dedication).
