# Supplementary material for: Effective image visualization for publications – a workflow using open access tools and concepts
Source: F1000Res. 2021 Feb 18;9:1373. Originally published 2020 Nov 26. [Version 2] doi: 10.12688/f1000research.27140.2 (PMC7931257; doi:10.12688/f1000research.27140.2)
Supplement: Peer review history

# Peer review: Reviewer response for version 2

**DOI:** 10.5256/f1000research.54504.r79893

**Published:** 2021-03-03

**Authors:** Georgina Fletcher, David Barry

**Affiliations:** 1 The Francis Crick Institute, London, UK; 2 BioImagingUK, Royal Microscopical Society, Oxford, UK

We have looked at Schmied and Jambor’s revisions and are satisfied that they have addressed all our points to a reasonable standard. We find that the article is much improved and will be a valuable community resource and thus recommend indexing.

 We have the following minor points:

- Step 2: References are made to Histograms A, B and C in Figure 3, but the histograms are not labelled accordingly in the figure.
- Figure 2: The Drosophila egg chambers have been removed but the text (“Testing of workflow”) still refers to them. Perhaps say “data not shown”?
- Figure 2B: I assume "color blind save" should read "color blind safe"?

Are the conclusions about the tool and its performance adequately supported by the findings presented in the article?

Yes

Is the rationale for developing the new software tool clearly explained?

Yes

Is the description of the software tool technically sound?

Yes

Are sufficient details of the code, methods and analysis (if applicable) provided to allow replication of the software development and its use by others?

Partly

Is sufficient information provided to allow interpretation of the expected output datasets and any results generated using the tool?

Partly

Reviewer Expertise:

Bioimage analysis (David Barry) and cell and developmental biology (Georgina Fletcher).

We confirm that we have read this submission and believe that we have an appropriate level of expertise to confirm that it is of an acceptable scientific standard.

# Peer review: Reviewer response for version 1

**DOI:** 10.5256/f1000research.29982.r75495

**Published:** 2020-12-14

**Authors:** Georgina Fletcher, David Barry

**Affiliations:** 1 BioImagingUK, Royal Microscopical Society, Oxford, UK; 2 The Francis Crick Institute, London, UK

**General Comments:**

 The authors present a suggested workflow to assist researchers in the life sciences in preparing images for presentation and/or publication. How images should be edited and/or adjusted in a suitable manner prior to submission to journals is certainly a source of confusion for many biologists, as they typically lack training in fields such as microscopy and image analysis, as noted by the authors. Therefore, a simple guide on what constitutes appropriate image adjustment would certainly be a valuable resource for the community. However, there are a number of technical points that require revision if the intended target audience (i.e those lacking a good understanding of image processing are to carry out the workflow correctly). There are also a number of revisions we would suggest to improve clarity and readability.

**Specific Points:**

- With the greatest of respect to the authors, the manuscript would benefit from proof-reading by a native English speaker.
- "Every day, around 2000 biomedical articles appear, 500 of which contain images." - a reference is needed to support this statement.
- It would be good to have more emphasis in the introduction on quality image acquisition being crucial to obtaining good images and that often processing afterwards will not make up for this. In particular, in terms of zoom, brightness/contrast, image alignment and treating all samples in the same way.

**Step 1:**

 Start with safely saving and storing raw images, and making a duplicate before opening it in FIJI. It would be helpful to explain image formats more fully (i.e why TIFF and not JPEG is preferred). It may be useful to add Image Compression to the Glossary terms. Probably worth mentioning Bio-Formats here to ensure correct reading of metadata. In addition, should "open" and "save" not be two different steps? It may also be worth mentioning that saving with Bio-Formats allows full metadata preservation (and permits lossless TIFF compression).

**Step 2:**

 There's an ambiguity here between preparing an image for publication/presentation and preparing an image for quantification - these are two different things and should be treated as distinct. In terms of contrast adjustment, it should perhaps be mentioned that a little saturation here is ok if it aids visualisation. Probably state that it’s good practice to avoid any auto buttons to ensure that all images are treated the same way as software packages can differ in what auto actually means. Explain either in the main text or the Glossary what a histogram and linear/non-linear adjustments are, and note what is acceptable for presentation but not quantification. Add to the last sentence “in the figure legend and the methods”.

**Step 3:**

 "Often further processing is necessary" - no processing has been performed as yet? This whole section is quite confusing and again, there's too much reference to quantification here - the purpose of the pipeline should be solely on adjustments necessary for publication. I would argue that image processing such as maximum intensity projection or Gaussian filtering should only be performed if the intention is to illustrate an analysis pipeline.

**Step 4:**

 Further reference to quantification. Image rotation does not reduce unnecessary information. Do you mean image resizing? Aligning specimens again would be best done whilst imaging. Explain why steps of 90 degrees rotation are acceptable, but any other angles bring up issues.

**Step 5:** Probably makes sense to crop before resizing?

**Step 6:**

 It’s also better for black and white printing to have grayscale images. Each channel separated and grayscale, with a composite (it’s in the cheat sheet, but it should be here too). Here it’s written that a black background is preferred, but in the cheat sheet it states that an inverted image gives the best contrast, please resolve.

**Step 7:**

 Mention that good annotations allow the reader to understand the results without reading any text?

**Results:** Reference to DPI is confusing. It should be explained that pixels-per-inch in the context of printing is distinct from microns-per-pixel in the context of image acquisition.

**Figure 2:** I'm not sure there's a need for Figure 2C? Why not just show the colour-blind friendly colour scheme in Fig 2B?

**Figure 3:**

- I'm not sure there's a need to reference update sites here?
- ”Tip” is misspelled throughout.
- Under "Open & Save", I would suggest always using Bio-Formats to open images.
- Under "Brightness & Contrast", I would remove all references to acquisition settings, instead referring to other publications on the matter ^1^^,^^2^.
- Also, I don't understand what is meant by "background not cropped"? I would expect all microscopy images to have a non-zero offset?
- I think the “Typical problems” needs further explanation in the text as a novice user may not understand the concepts from the diagrams alone. Please explain what is being looked at in the histograms.
- Remove references to image processing - why would the images be processed if the intention is purely for illustration?
- Under "Rotating & Resizing", it should be mentioned that rotating by anything other than a multiple of 90 degrees should be discouraged as this will lead to interpolation.
- Under "Color", the simultaneous reference to viewing images as composites and splitting channels is confusing. I think there's probably a little too much information under this heading and certain things could be dispensed with, such as inverting for better visibility (or perhaps just mention it in the text).
- Under "Annotate", there should no need for Analyze > Set Scale if the metadata has already been verified.

**Figure 4:**

- Examples images used under "Magnification" and "Zoom, Insets" are not great.
- I feel like Figures 3 and 4 could be combined into one single cheat sheet?

**Reference 30** cites this paper but without its first author – is this correct?

Are the conclusions about the tool and its performance adequately supported by the findings presented in the article?

Yes

Is the rationale for developing the new software tool clearly explained?

Yes

Is the description of the software tool technically sound?

Yes

Are sufficient details of the code, methods and analysis (if applicable) provided to allow replication of the software development and its use by others?

Partly

Is sufficient information provided to allow interpretation of the expected output datasets and any results generated using the tool?

Partly

Reviewer Expertise:

Bioimage analysis (David Barry) and cell and developmental biology (Georgina Fletcher)

We confirm that we have read this submission and believe that we have an appropriate level of expertise to confirm that it is of an acceptable scientific standard, however we have significant reservations, as outlined above.

## References

[1] : Tutorial: guidance for quantitative confocal microscopy. *Nat Protoc*.15(5) : 10.1038/s41596-020-0313-9 1585-1611 10.1038/s41596-020-0313-9 32235926

[2] : Designing a rigorous microscopy experiment: Validating methods and avoiding bias. *Journal of Cell Biology*.2019;218(5) : 10.1083/jcb.201812109 1452-1466 10.1083/jcb.201812109 PMC650488630894402

# Response

**Published:** 2021-02-04

**Authors:** Helena Jambor

**Affiliations:** Technische Universität Dresden, Germany

We thank the reviewers for their constructive suggestions, which have substantially improved the manuscript and cheat sheets. Below we respond to the reviewers comments ( *in italics*) in detail and describe the modifications we implemented in text and figures/cheat sheets.

***Specific Points:***

- *With the greatest of respect to the authors, the manuscript would benefit from proof-reading by a native English speaker.*

**RESPONSE**: We indeed aren’t native speakers and have now convinced an American partner to once more proof-read, with greater care, the manuscript.

  

- *"Every day, around 2000 biomedical articles appear, 500 of which contain images." - a reference is needed to support this statement.*

**RESPONSE: **We agree with the reviewers that this sentence warrants a citation indicating the source of these numbers. We have now amended the sentence to state:

 “Every year, about 800,000 articles are indexed at Pubmed ( https://www.nlm.nih.gov/bsd/index_stats_comp.html) of which 25% contain images (Lee et al. 2018); this amounts to about 500 new articles with images every single day.”

  

- *It would be good to have more emphasis in the introduction on quality image acquisition being crucial to obtaining good images and that often processing afterwards will not make up for this. In particular, in terms of zoom, brightness/contrast, image alignment and treating all samples in the same way.*

**RESPONSE**: We agree that image acquisition is always the critical step in producing good images. The microscopy community has extensively covered many aspects of going from specimen to image; while the message might not have reached everyone, resources and guidelines on image acquisition are plentiful. On the other hand, we see a lack in resources to making such images legible for audiences, and this is what our main focus is; We have now expanded the first sentence of the ‘Methods’ to emphasize further the importance of good image quality before setting out to create publishable image figures. We now say:

 “Obtaining high quality bioimages starts with specimen preparation such as fixation, labelling and clearing. To acquire and resolve the biological structure of interest, choose a microscopy system with an objective lens that allows suitable resolution, optical sectioning and spatial sampling. It is vital to sample intensity information properly by choosing a sufficient bit depth and avoiding saturation of high intensities. If the microscope-system allows changing the detector offset, low intensities should not be cut off. Rather than down sampling and cropping the image data, choose an appropriate magnification. When possible, align or rotate the sample to avoid image rotations. For comparison of image data, sample preparation and image acquisition settings need to be the same [12-19]. “

***Step 1:***

*Start with safely saving and storing raw images, and making a duplicate before opening it in FIJI. It would be helpful to explain image formats more fully (i.e why TIFF and not JPEG is preferred). It may be useful to add Image Compression to the Glossary terms. Probably worth mentioning Bio-Formats here to ensure correct reading of metadata. In addition, should "open" and "save" not be two different steps? It may also be worth mentioning that saving with Bio-Formats allows full metadata preservation (and permits lossless TIFF compression).*

**RESPONSE**: We thank the reviewers for these suggestions.

 - Indeed, in the cheat sheet the first suggested step is making a duplicate of the raw image and we now include this in the manuscript too.

 - We indeed now expanded the glossary to explain image file-formats including TIFF, JPEG, PNG, Bio-Formats and Image compression, a point also raised by reviewer 1.

 - the section is named ‘open & save’ to highlight the first point of the reviewer, that the first step after opening an image should be to make a copy, and also highlight upfront the consequence of certain file formats.

 Step 1 now says:

 “Duplicate the raw image to retain the original, untouched image as raw data and only process the duplicate. Load image-duplicate into Fiji and make sure metadata (see Table 1: Glossary), such as the scale, are correct. When possible use the Bio-Formats plugin for import, as this reads key image metadata (e.g. scale) automatically along with the image [22].

 When processing is complete, several options exist (see glossary): saving images in TIFF format preserves the entire information. TIFF files however can rarely be properly used in programs for figure assembly (e.g. Inkscape, PowerPoint). For image presentation (figures, slides, online), save images in PNG format, which irreversibly merges the image with annotations, permanently applies brightness/contrast settings, and saves multiple channels as 24-bit RGB image. Another common image presentation file format is JPEG, which should be rarely used due to its lossy compression [19]. Beware of incorrect or unintentional bit depth conversions [23].”

***Step 2:***

*There's an ambiguity here between preparing an image for publication/presentation and preparing an image for quantification - these are two different things and should be treated as distinct. In terms of contrast adjustment, it should perhaps be mentioned that a little saturation here is ok if it aids visualisation. Probably state that it’s good practice to avoid any auto buttons to ensure that all images are treated the same way as software packages can differ in what auto actually means. Explain either in the main text or the Glossary what a histogram and linear/non-linear adjustments are, and note what is acceptable for presentation but not quantification. Add to the last sentence “in the figure legend and the methods”.*

**RESPONSE**: We thank the reviewers for these points and have incorporated them into a new Step 2 description, which also incorporates a suggestion by reviewer 1. Intensity adjustment (i.e. brightness and contrast) and 2 examples of nonlinear adjustments are now also incorporated here and the glossary. We added image histograms as a topic to the glossary and mention that one can analyze image intensity sampling problems using the image histogram. The new step 2 now says:

 “Images with a large gray value range may appear black when opening them in FIJI [12]. To properly display such data for the purpose of presentation/communication [24], adjust the brightness and contrast. For comparisons of intensities across images, it is recommended to use the same fixed intensity values (‘set’). For adjustments, avoid auto-buttons as, depending on the software packages the underlying code may differ, resulting in display differences. Linear intensity adjustment is acceptable, as long as key features are not obscured and minimal background signal is still visible to provide audiences with a sense for signal specificity. Entirely eliminating the background signal, or completely ‘clipping’ high intensities, is misleading (see also [10, 19, 25]). Some saturated pixels in the image are acceptable, if this helps the visualization. To identify problems with intensity sampling, or seeing if the image has been processed, the image histogram can be used to show its gray value distribution (Fig. 3). Briefly, good unprocessed images should have some offset in the low intensity range (Fig. 3: Histogram A). The distribution should not be cut off in the high range (Fig. 3: Histogram B) and the range should be continuous (Fig. 3: Histogram C) 

 Non-linear adjustments of brightness and contrast, for example histogram equalizations or gamma correction [19, 26] must be explained in both figure legend and method section [19, 26, 27]. Miura and Nørrelykke nicely describe why intensity adjustments (linear and non-linear) must be applied with special caution when images have already been pre-processed, e.g. cropped [21]. Once images have been adjusted, ‘apply’ and ‘save as PNG’ irreversibly change the intensity range, which makes images unsuitable for intensity measurements.”

***Step 3:***

*"Often further processing is necessary" - no processing has been performed as yet? This whole section is quite confusing and again, there's too much reference to quantification here - the purpose of the pipeline should be solely on adjustments necessary for publication. I would argue that image processing such as maximum intensity projection or Gaussian filtering should only be performed if the intention is to illustrate an analysis pipeline.*

**RESPONSE**: We thank the reviewers to help us clarify this section. Our intention was to leave a buffer zone in the work process where readers can then perform their specific processing   - as these vary dramatically, we cannot possibly start commenting. We are also of the opinion that carefully applied image processing in specific and informed instances is unavoidable for proper image visualization. For instance in advanced systems, reconstruction has to be performed to visualize the biological structure of interest. Also the important information is the actual signal of the biological specimen, which can be degraded, even with highly optimized acquisition, by imaging artefacts such as noise and blur. Here appropriate image processing, made transparent in the publication and guaranteeing the access to the raw image data can promote visualization (Cromey 2010, 2013 Jonkman et al. 2020). It is really crucial that researchers are aware that appropriately used image processing can be fine in specific well defined circumstances, under the condition that necessary information is provided for reproduction AND are not used as a replacement for optimized imaging. We made this section clearer by properly specifying and citing examples for standard or advanced image processing with the aim of allowing or promoting visualization. Also we strongly emphasizes the key points that methods need to be used only in specific conditions, are not replacement for good imaging and need to be made transparent.

 “Depending on your specific scientific question and goal, further image processing may be necessary for image visualization. For instance, advanced systems such as lightsheet microscopy require extensive image processing workflows to obtain a reconstructed volume of the biological specimen for visualization [28-32]. Large 3D volumes of data are also hard to visualize in two dimensional figures and require the use of projection or rendering [33]. Finally, microscopy systems also add artefacts (noise, blur), which image processing using linear filters [13] and deconvolution [34, 35] can help to reduce. Any processing for representing the image data needs to be carefully applied where necessary and is no replacement for an optimized imaging setup [12-18]. The processing needs to be clearly stated in the methods section, advanced or non-linear adjustments also in the figure legends [13, 19].”

***Step 4:***

*Further reference to quantification. Image rotation does not reduce unnecessary information. Do you mean image resizing? Aligning specimens again would be best done whilst imaging. Explain why steps of 90 degrees rotation are acceptable, but any other angles bring up issues. **Step 5: **Probably makes sense to crop before resizing?*

**RESPONSE**: We thank the reviewers for requiring us to disentangle image rotation and resizing, we now have included Step 4: Image rotation and Step 5: Cropping and resizing. We have also updated Figure 1 accordingly. In the new section 4 we also explain why rotation is necessary and the specific case of 90-deg rotation (which depends on the software used if it indeed is lossless rotation or not). The new Steps 4 and 5 say:

 “Step 4: Image rotation

 Image rotations are sometimes necessary to compare image content properly. For instance, when comparing specimens, it helps to align them in the same anatomical orientation. Image rotations however result in a redistribution of the intensity values within the fixed image pixel grid: for rotations by less than 90 degrees, new intensity values are computed by interpolation, and thus information is lost (Fig. 3). For rotations in multiples of 90-degree steps, pixels can be reordered rather than interpolated, however this depends on the specific implementation of the rotation algorithm (Fig. 3). Loss of information by image rotation may be acceptable for image visualizations, however all image quantifications should be done beforehand [19, 26].

 Step 5: Cropping & resizing

 Often larger fields-of-view are captured on the microscope than are required in the figure. Cropping is then not only permissible, it is necessary to focus the reader on the relevant result. In contrast, it is not ethical to crop out data that would change the interpretation of the experiment, or to “cherry-pick” data [10, 19, 26]. We discourage adjusting the intensity of individual crops especially for comparisons [21].  When a larger field of view and a magnification of detail (‘inset’) need to be shown side-by-side, indicate inset position in the original image. Adjust the size of the image in the figure preparation software, not during image processing: Image size adjustments by upsampling or downsampling an image, requires interpolation and thus may degrade image quality.”

***Step 6:***

*It’s also better for black and white printing to have grayscale images. Each channel separated and grayscale, with a composite (it’s in the cheat sheet, but it should be here too). Here it’s written that a black background is preferred, but in the cheat sheet it states that an inverted image gives the best contrast, please resolve.*

**RESPONSE**: We thank the reviewers for catching our inconsistency and have adapted to the text to fit the cheat sheet. The first section of step 6 now says:

 “Step 6: Color

 In fluorescence microscopy, cameras usually capture each wavelength (channel) with a separate grayscale image. Here, no signal is shown as black, and intensities of the fluorescent signal are displayed in steps of grey values with saturated pixels shown in white. When only one fluorophore/wavelength/channel is shown in a figure, grayscale, which has the best contrast, is favorable. Consider also inverting the grayscale images as human brightness perception is logarithmic and can best differentiate bright areas [27]. Inverted grayscale images are also printer-friendly and have better visibility on a white page/slide. To visualize several channels of a specimen (e.g. colocalization studies), encode channels with different colors. A look-up table (LUT) determines how gray values are translated into a color value. Additionally, we often sometimes see the use of false color LUTs for visualizing image data; when used improperly, false color LUTs can be highly misleading [27] and therefore should be explicitly mentioned in methods and figure legends.”

***Step 7:***

*Mention that good annotations allow the reader to understand the results without reading any text?*

**RESPONSE**: We thank reviewers for this suggestion and have added it to the adapted Step 7, which now reads:

 “Images represent physical dimensions and can depict different scales ranging from nanometer to millimeter, which is often not obvious [36]. Adding scale information, ideally a scale bar with dimension, onto or next to the image, therefore is essential for self-explanatory figures. Also annotate what each color and symbol represents in an image, again best in the image itself or next to it. The aim is to provide sufficient information to the reader to understand the presented result at a glance. Ensure that scale bar, dimensions and annotations are legible in the final figure to be published; it may be more time efficient to adjust scale bar and add dimensions/annotations in the figure preparation software (e.g. as described here [37]).”

***Results:***

*Reference to DPI is confusing. It should be explained that pixels-per-inch in the context of printing is distinct from microns-per-pixel in the context of image acquisition.*

**RESPONSE**: We thank reviewers for noting our imprecise description - and indeed, as we write in the section, feedback from colleagues helps!  We stayed with using dots per inch as a term instead of pixel-per-inch (screen res) since for figure preparation the output of many programs (Illustrator, Photoshop) as well as author guidelines usually specify this term. The section in the results now says:

 “Figure resolution is usually referred to as dots per inch (DPI). For an ‘unpixelated’ display of microscopy images in an electronic publication, publishers require 300 DPI images in RGB color mode. (Note that the dots-per-inch do not correspond to the physical dimension of the microscopy object and scale bar but solely refer to image size in print or on the screen). This workflow is iterative and feedback from colleagues helps to identify possible hurdles.”

***Figure 2:***

*I'm not sure there's a need for Figure 2C? Why not just show the colour-blind friendly colour scheme in Fig 2B?*

**RESPONSE**: We thank reviewers for this suggestion and based on their suggestion have simplified Figure 2.

***Figure 3:***

- *I'm not sure there's a need to reference update sites here?*

**RESPONSE**: We agree and have now removed the comment from the cheat sheet. 

- *”Tip” is misspelled throughout.*

**RESPONSE**: We thank the reviewers (and several readers on twitter) for catching this mistake and have updated it in Figure 3 and 4.

- *Under "Open & Save", I would suggest always using Bio-Formats to open images.*

**RESPONSE**: We now specify Bio-Formats as the recommended opening and specify in the text that the Bio-Formats plugin is metadata friendly.

  

- *Under "Brightness & Contrast", I would remove all references to acquisition settings, instead referring to other publications on the matter 1,2.*

**RESPONSE**: We agree and remove this section of the TIP instead refer to the publications for further information about image acquisition.

- *Also, I don't understand what is meant by "background not cropped"? I would expect all microscopy images to have a non-zero offset?*

**RESPONSE**: This is exactly what we wanted to express, since in our practice it can happen that people incorrectly set a detector offset cutting of low values. We agree that this was not written clearly. We adjusted the text next to the Figure to indicate how an acceptable histogram of a raw image could like and that the other histograms represent that the image was altered or is problematic.

- *I think the “Typical problems” needs further explanation in the text as a novice user may not understand the concepts from the diagrams alone. Please explain what is being looked at in the histograms.*

**RESPONSE**: We agree and have added an explanation in the text.

 “Briefly, good unprocessed images should have some offset in the low intensity range (Fig. 3: Histogram A). The distribution should not be cut off in the high range (Fig. 3: Histogram B) and the range should be continuous (Fig. 3: Histogram C).” 

  

- *Remove references to image processing - why would the images be processed if the intention is purely for illustration?*

**RESPONSE**: In principle we agree that as little processing as necessary should be applied to images for visualizations. However, as we tried to lay out in the rebuttal (Step 3 - Image processing) in some instances image processing cannot be avoided and for some instances of microscopy artefact it might also be beneficial for visualization (Cromey 2010, 2013, Jonkman et al. 2020). Thus, image processing done correctly and specified transparently in the methods as well as figure legends can be in our opinion permissible.

- *Under "Rotating & Resizing", it should be mentioned that rotating by anything other than a multiple of 90 degrees should be discouraged as this will lead to interpolation.*

**RESPONSE**: We agree and added an explanatory sentence.

- *Under "Color", the simultaneous reference to viewing images as composites and splitting channels is confusing. I think there's probably a little too much information under this heading and certain things could be dispensed with, such as inverting for better visibility (or perhaps just mention it in the text).*

**RESPONSE**: We changed the place for showing the tip as indeed this is not relevant for the very first command.

- *Under "Annotate", there should no need for Analyze > Set Scale if the metadata has already been verified.*

**RESPONSE**: We agree that if the cheat sheet is linearly read, this information is not strictly required at that specific stage. We however expect that users will also read the cheat sheet to look up a specific action or possibly use drag-and-drop for file import. We therefore feel a slight redundancy does not do any harm and has the benefit that users can double check the information.

***Figure 4:***

- *Examples images used under "Magnification" and "Zoom, Insets" are not great.*

**RESPONSE**: We thank reviewers for this comment and have updated the images.

  

- *I feel like Figures 3 and 4 could be combined into one single cheat sheet?*

**RESPONSE**: We’d rather prefer to have two individual Figures in the manuscript that can be individually referenced. The first cheat sheet serves as a general template independent of a specific implementation and is thus crucial for people that use any of the many other open source or proprietary software solutions. The second cheat sheet implements these general principles with FIJI. Thus, both cheat sheets are crucial and can work stand-alone.  Also, we hope that readers will print each figure as an A4/Letter cheat sheet. Combining the figures would inevitably result in too small printed versions.

 We changed the text in the Results section to explain our intentions better:

 “The workflow steps and accompanying suggestions for image presentation are available as accessible “cheat sheets” (Fig. 3, 4) for wide distribution and adoption to more specific needs. Our workflow is based on the open source software Fiji (Fig. 3), but its principles are applicable to other software (Fig. 4).”

*Reference 30*

*cites this paper but without its first author – is this correct?*

**RESPONSE**: Yes, as only one author is registered with OSF. In the new version the second author also signed up for OSF and is now listed as co-author.

# Peer review: Reviewer response for version 1

**DOI:** 10.5256/f1000research.29982.r75494

**Published:** 2020-12-09

**Authors:** Guillaume Jacquemet

**Affiliations:** 1 Turku Bioscience Centre, University of Turku, Turku, Finland

In this article, Christopher Schmied and Helena Klara Jambor provide an excellent overview of the steps and concept that should be taken in consideration when preparing figures containing microscopy images. The article is of very high quality and will be very useful for students and seasoned microscopists alike. I will certainly make it a mandatory read for my lab members.

 Below are several comments that came to mind while reading the manuscript that the authors may want (or not) to consider. Several of them are perhaps out of the scope of this work but I will write them down in any case for the authors' consideration.

1. While the manuscript is intended to educate and improve image display in figures, it may be useful to add a disclaimer that images are illustrations and should be associated with a quantification of the phenomenon displayed.
2. In the manuscript, the authors discuss changing the brightness contrast to improve the images visually. But other manipulations such as changing the gamma or the colour balance should probably also be mentioned. While the appropriateness of these manipulations is often debated for scientific images, it would be useful to educate the readers about their existence and their pitfall. Especially since the correct gamma to use depends on the display used to visualise the images.
3. On a similar note, it may be useful to inform that performing "background clipping" may also not be an appropriate image manipulation.
4. On a similar note, it may be useful to add a warning message in the LUT section indicating that not all LUT display the image intensities linearly.
5. It may be useful to explain the benefit of using PNG over TIFF images when preparing figures.
6. I would personally not recommend annotating and cropping images directly in ImageJ but rather do these operations in the software used to build the figures themselves. This ensure that the annotations are easily changed (font, size) and are scalable (when exported to PDF). But that is likely a personal preference.
7. It would be useful to list a few software solution that can be used to create the figures. For instance if done directly in ImageJ, FigureJ is a popular option. I use Inkscape to build figure panels. In this context, it would be useful to indicate that software may modify images when importing them and that this should be avoided (image artefacts due to compression or upscaling).

Are the conclusions about the tool and its performance adequately supported by the findings presented in the article?

Yes

Is the rationale for developing the new software tool clearly explained?

Yes

Is the description of the software tool technically sound?

Yes

Are sufficient details of the code, methods and analysis (if applicable) provided to allow replication of the software development and its use by others?

Yes

Is sufficient information provided to allow interpretation of the expected output datasets and any results generated using the tool?

Yes

Reviewer Expertise:

Cell biology

I confirm that I have read this submission and believe that I have an appropriate level of expertise to confirm that it is of an acceptable scientific standard.

# Response

**Published:** 2021-02-04

**Authors:** Helena Jambor

**Affiliations:** Technische Universität Dresden, Germany

We thank Guillaume Jacquemet for these constructive suggestions to improve our paper. The reviwers comments are shown in *italic text. *Our detailed responses and an indication of the implemented modifications to the manuscripts are below.

*1) While the manuscript is intended to educate and improve image display in figures, it may be useful to add a disclaimer that images are illustrations and should be associated with a quantification of the phenomenon displayed.*

**RESPONSE**: We agree that qualitative images and quantifiable data from images are distinct and have now included a sentence in the methods section, which also refers to a workflow for reproducible image quantification:

 “Images are quantitative data. While image visualizations allow qualitative assessments, it is important to accompany them with quantitative measurements and appropriate statistical analysis. This workflow strictly addresses the image processing necessary for presentations and figures. Images prepared for presentation (e.g. 8-bit, RGB) are unsuitable for subsequent quantification such as intensity measurements. We therefore recommend separating image quantification and visualization workflows. Finally, documentation of any imaging and image processing workflows is key for reproducibility [21].”

*2) In the manuscript, the authors discuss changing the brightness contrast to improve the images visually. But other manipulations such as changing the gamma or the colour balance should probably also be mentioned. While the appropriateness of these manipulations is often debated for scientific images, it would be useful to educate the readers about their existence and their pitfall. Especially since the correct gamma to use depends on the display used to visualise the images.*

 and

 3) *On a similar note, it may be useful to inform that performing "background clipping" may also not be an appropriate image manipulation.*

**RESPONSE**: Excellent point. We already mention in step 2 that ‘ *non-linear adjustments, .. e.g. gamma correction need to be explained*’ and have now expanded the section to indicate this more clearly.

 We also agree with the reviewer that background clipping, especially when done excessively, may be misleading audiences. As both points are prominently highlighted in the CSE’s White Paper [Ref 9] and in the milestone blog by the Journal of Cell Biology, we have clearly referenced these resources for further guide audiences. The section now reads as follows (also integrating comments by reviewers 2):

 “Images with a large gray value range may appear black when opening them in FIJI [12]. To properly display such data for the purpose of presentation/communication [24], adjust the brightness and contrast. For comparisons of intensities across images, it is recommended to use the same fixed intensity values (‘set’). For adjustments, avoid auto-buttons as, depending on the software packages the underlying code may differ, resulting in display differences. Linear intensity adjustment is acceptable, as long as key features are not obscured and minimal background signal is still visible to provide audiences with a sense for signal specificity. Entirely eliminating the background signal, or completely ‘clipping’ high intensities, is misleading (see also [10, 19, 25]). Some saturated pixels in the image are acceptable, if this helps the visualization. To identify problems with intensity sampling, or seeing if the image has been processed, the image histogram can be used to show its gray value distribution (Fig. 3). Briefly, good unprocessed images should have some offset in the low intensity range (Fig. 3: Histogram A). The distribution should not be cut off in the high range (Fig. 3: Histogram B) and the range should be continuous (Fig. 3: Histogram C) “

*4) On a similar note, it may be useful to add a warning message in the LUT section indicating that not all LUT display the image intensities linearly.*

**RESPONSE**: We thank the reviewer for bringing up this interesting point. After educating ourselves on this topic, we have included a note in the paper and cheat sheet and referenced a useful resource. We now include the sentence below in Step 6 and also expanded the glossary.

 “Additionally, we often sometimes see the use of false color LUTs for visualizing image data; when used improperly, false color LUTs can be highly misleading [26] and therefore should be explicitly mentioned in methods and figure legends.”

*5) It may be useful to explain the benefit of using PNG over TIFF images when preparing figures.*

**RESPONSE**: We agree with the reviewers to include a statement of the benefit of PNG file format for publications and, on that note, also contrast it with the JPEG format. We now provide more details in the relevant sentence of ‘Step 1’ and also included a section of file formats in the glossary.

 “When processing is complete, several options exist (see glossary): saving images in TIFF format preserves the entire information. TIFF files however can rarely be properly used in programs for figure assembly (e.g. Inkscape, PowerPoint). For image presentation (figures, slides, online), save images in PNG format, which irreversibly merges the image with annotations, permanently applies brightness/contrast settings, and saves multiple channels as 24-bit RGB image. Another common image presentation file format is JPEG, which should be rarely used due to its lossy compression [19]. Beware of incorrect or unintentional bit -depth conversions [23].”

*6) I would personally not recommend annotating and cropping images directly in ImageJ but rather do these operations in the software used to build the figures themselves. This ensure that the annotations are easily changed (font, size) and are scalable (when exported to PDF). But that is likely a personal preference.*

**RESPONSE: **We personally agree with the reviewer and indeed the authors themselves produce legible scale bars with the described procedure (see our post on theNode). We also already mention this as a TIP in the figure 4/Cheat sheet for publishing images. We are however also aware opponents of this method, who fear that it may result in improper scales. We have now amended ‘step 7’ in the paper and added a cautionary note in the FIJI cheat sheet (Fig. 3). Step 7 now says:

 “Images represent physical dimensions and can depict different scales ranging from nanometer to millimeter, which is often not obvious [36]. Adding scale information, ideally a scale bar with dimension, onto or next to the image, therefore is essential for self-explanatory figures. Also annotate what each color and symbol represents in an image, again best in the image itself or next to it. The aim is to provide sufficient information to the reader to understand the presented result at a glance. Ensure that scale bar, dimensions and annotations are legible in the final figure to be published; it may be more time efficient to adjust scale bar and add dimensions/annotations in the figure preparation software (e.g. as described here [37]).”

*7) It would be useful to list a few software solution that can be used to create the figures. For instance if done directly in ImageJ, FigureJ is a popular option. I use Inkscape to build figure panels. In this context, it would be useful to indicate that software may modify images when importing them and that this should be avoided (image artefacts due to compression or upscaling).*

**RESPONSE: **We agree with the reviewer that audiences appreciate suggestions for open source software and have expanded the results section of the manuscript, where we already referenced, without explicitly naming, FigureJ. The second paragraph of the results section now states:

 “After completing the workflow, images may be assembled for publication and legends added [41]. Layouting images on a page can be done with design software such as the free and open source Inkscape (https://inkscape.org) or the proprietary Adobe Illustrator. Several options also exist to prepare publication-ready figures directly in ImageJ/FIJI, for example ScientiFig and FigureJ [42, 43].”
